# Treatment Outcome and Risk Factors of Adult Newly Diagnosed Epilepsy: A Prospective Hospital-Based Study in Northeast China

**DOI:** 10.3389/fneur.2021.747958

**Published:** 2021-10-28

**Authors:** Nan Li, Jing Li, Yanyan Chen, Chaojia Chu, Weihong Lin

**Affiliations:** ^1^DDepartment of Neurology, The First Hospital of Jilin University, Changchun, China; ^2^Department of Neuroelectrophysiology, Changchun Six Hospital, Changchun, China

**Keywords:** drug resistant epilepsy, antiseizure medication, risk factors, adult, newly diagnosed epilepsy

## Abstract

**Objective:** The study was conducted to summarize the treatment outcomes of newly diagnosed epilepsy (NDE) and analyse the risk factors for refractory epilepsy (RE) in Northeast China.

**Methods:** A total of 466 adult patients with NDE were consecutively enrolled in this programme. Clinical data were collected at baseline and each follow-up. Several scales concerning recognition and mood were also completed at the first visit.

**Results:** Seizure-free status was achieved by 52% (*n* = 244) of the patients; however, 15% (*n* = 68) manifested RE. A total of 286 (61%) patients continued with the first ASM as monotherapy, among which 186 (40%) patients became seizure-free. Fifteen (22%) patients with RE became seizure-free following ASM adjustment and 34 patients (14%) had breakthrough seizures after being classified as seizure-free. One patient developed RE after attaining seizure-free status. Breakthrough seizures during the first expected interictal interval [Odds ratio (OR) = 5.81, 95% CI: 2.70–12.50], high seizure frequency at baseline (OR = 1.24, 95% CI: 1.04–1.49), younger age of onset (OR = 1.42, 95% CI: 1.12–1.79), and male sex (OR = 2.64, 95% CI: 1.26–5.53) were risk factors for RE.

**Significance:** Treatment outcomes of the majority of NDE cases are good. New risk factors could help physicians more promptly and accurately identify patients who are likely to develop RE. Seizure-free state is not long enough to commence the withdrawal of ASMs. RE is not permanent and seizure-free may be achieved subsequently by appropriate drug adjustment.

## Introduction

Epilepsy is a serious neurological disorder that affects more than 70 million people worldwide, ranging from neonates to older adults (1). In China, the number of patients with epilepsy was ~10 million in 2015 (2). Pharmacotherapy is the first choice for controlling epileptic seizures, and the majority of them could be controlled by currently available antiseizure medication (ASM). Refractory epilepsy (RE) is one of the most serious conditions, which affects 30–40% of people with epilepsy (3, 4). After years of multi-drug treatment with limited efficacy, patients with RE face great financial burden and mental pressure that seriously affect their quality of life. In this situation, making a precise diagnosis of RE is critical and would give a chance for appropriate subsequent treatments, such as neurostimulation and surgery. In previous studies (5–7), the diagnostic criteria for RE were inconsistent; thus, it is difficult to compare the conclusions across them. To set up explicit and practical criteria, the International League Against Epilepsy (ILAE) published a new definition of RE (8). That is, the minimum criteria for defining RE, ensuring that less time was wasted in inappropriate pharmacological therapy, thereby improving patient care. However, the definition has not been widely applied to the epidemiologic studies. Finding risk factors according to the new definition could help the physicians more promptly and accurately identify patients who are likely to develop RE.

This study consecutively enrolled patients with newly diagnosed epilepsy (NDE) at the Epilepsy Diagnosis and Treatment Center of the First Hospital of Jilin University, which is one of the biggest general hospitals in Jilin province, China. We summarized the treatment outcomes of NDE and analyzed the risk factors of RE in Northeast China.

## Materials and Methods

### Patient Recruitment

Patients visiting the Epilepsy Diagnosis and Treatment Center of the First Hospital of Jilin University were screened, and the adult patients who were newly diagnosed with epilepsy were consecutively enrolled in this programme between June 2015 and November 2019, and followed up until December 2020.

The definitions of epilepsy, the classification of seizure, and epileptic syndrome conformed to the diagnostic criteria published by ILAE (9–11). RE is defined as the failure of two tolerated and appropriate ASMs (whether monotherapy or in combination) to achieve sustained seizure-free state (8). The 50% defined daily dose (50% DDD) is considered as the “adequate dose” of each ASM (12). When patients are free from all seizures, including aura, for three times the interictal interval or 1 year (whichever is longer), they can be classified as seizure-free (8, 13). If the two abovementioned definitions cannot be satisfied, the outcome is designated as undetermined. The definition of a patient with NDE used in this study is a person with confirmed epilepsy who had not been diagnosed specifically with epilepsy or treated with ASMs previously.

### Study Procedure

At their first visit, all the participants underwent a thorough clinical and laboratory investigation, including a 24-h video electroencephalogram (EEG) and 3.0-T high-resolution brain magnetic resonance imaging (MRI). The patients were administered an ASM following the 2012 guidelines of the National Institute for Health and Clinical Excellence (14), starting at a low dose. If the patients with NDE agreed to participate in the programme and signed an informed consent form, a baseline file was completed, which contained demographic, symptomatic and etiologic data, as well as the results of a systematic physical examination, an EEG, and an MRI. The symptomatic data were collected by interviews with the patients or the witnesses to seizure. Participants were then asked to complete a series of scales, including the Montreal cognitive assessment (MOCA), the Generalized Anxiety Disorder 7-item Scale (GAD-7), and the Chinese version of the Neurological Disorders Depression Inventory for Epilepsy (c-NDDI-E), to estimate their cognitive function and mood.

The patients enrolled in the programme were called back for a follow-up visit for treatment adjustments at 1, 3, and 6 months following the treatment and every 6 months thereafter. In cases of seizure recurrence between scheduled appointments, the patient could visit the specialist epilepsy clinics. The second ASM was considered when the first one was ineffective or the patient had intolerable side effects. At every scheduled visit, a follow-up file was completed for all patients, which recorded the patients' seizure types and frequency, the doses of the ASMs administered, and any adverse effects. If a face-to-face visit was inconvenient, the follow-up file would be completed by physicians based on the interviews with patients or caregivers by telephone. Instances of patients withdrawing the ASMs without medical advice were defined as poor compliance. Patients were excluded if the follow-up periods were <12 months. The ASMs were gradually reduced and stopped if the patients had no breakthrough seizure for at least 3 years and the repeated EEG was normal.

### Statistical Analyses

Student's *t*-test, analysis of variance (ANOVA), Pearson's chi-squared test, the rank-sum test, and Fisher's exact test were used to compare continuous and categorical variables. A survival (Kaplan-Meier) analysis was often used to visually summarize time-to-event data and Log-rank was used to estimate the difference between the groups. Cox regression model analysis was applied to identify the risk factors for retention of the first ASM. Logistic regression was used to analyse the risk factors of RE.

Values for continuous variables are expressed as mean ± standard deviation (SD), and values for categorical variables are expressed as frequencies (%). All *p*-values were from two-tailed tests. *P* < 0.05 was considered to indicate statistical significance. The data were inputted by EpiData software (The EpiData Association, Odense, Denmark) and were subsequently analyzed using SPSS for Windows, Version 24.0 (SPSS Inc., Chicago, IL, USA).

### Ethical Approval

The protocol for this study was approved by the Ethics Committee of the First Hospital of Jilin University [the approval number: 2017-326] and was performed in accordance with the ethical standards laid down in the 1964 Declaration of Helsinki and its later amendments. Each enrolled patient provided a signed informed consent form before the study began.

## Result

### Demographic Information

**Figure 1:**
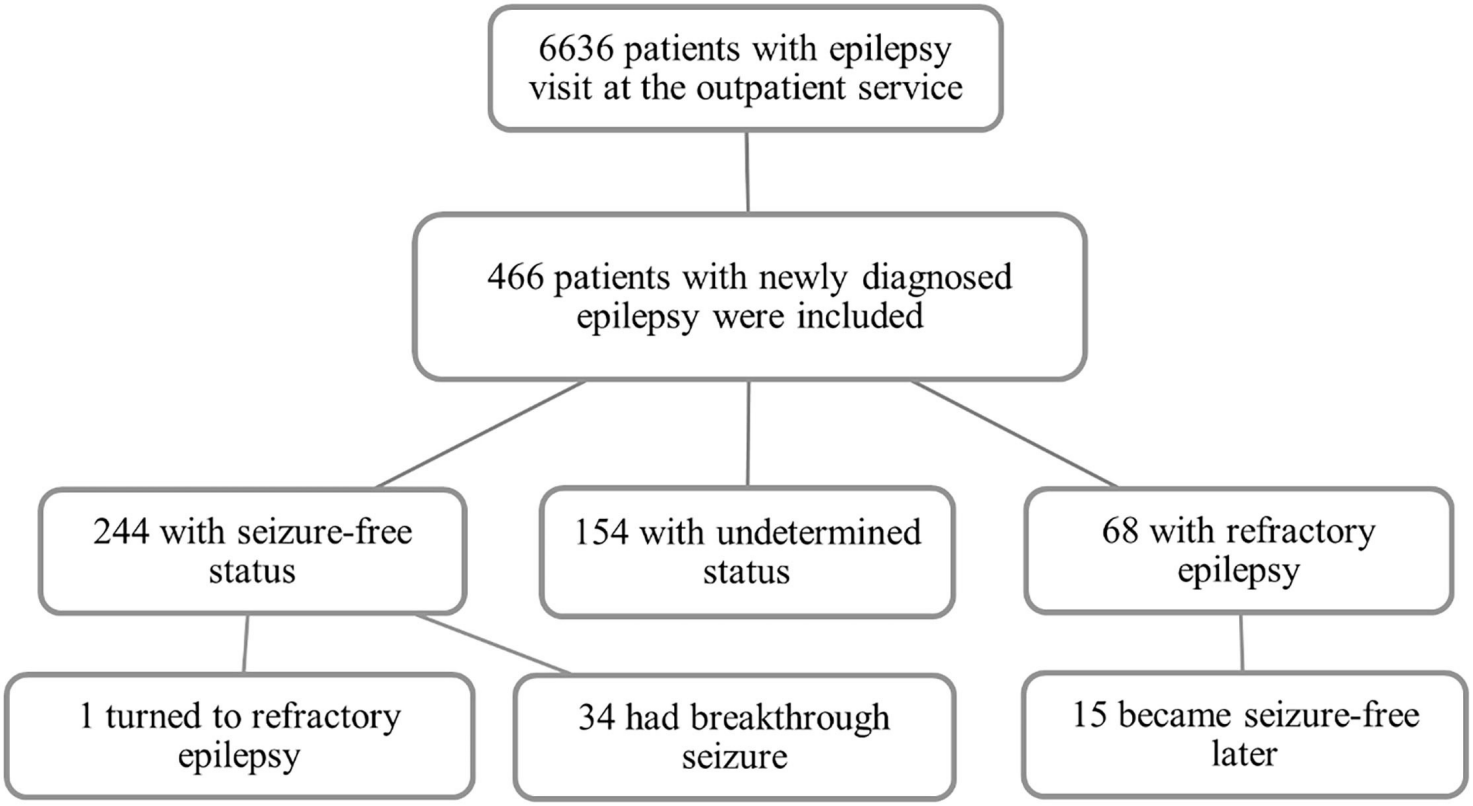
Flow diagram of the study.

A total of 6,636 people with epilepsy (PWE) who visited the Epilepsy Diagnosis and Treatment Center of the First Hospital of Jilin University were screened, and 466 patients were diagnosed as NDE and enrolled in the programme. The demographic information is shown in Table 1. The median follow-up time was 24 (range, 12–48) months. After treatment adjustments based on the responses to ASMs, 52% (*n* = 244) of the patients achieved seizure-free status; however, 15% (*n* = 68) were diagnosed as RE. The others (33%, *n* = 154) were undetermined (Figure 1). The median duration of treatment before arriving at RE and seizure-free status were 12 (range, 3–36) months and 12 (range, 12–36) months, respectively. About 74% (*n* = 50) of the patients required at least 12 months before being diagnosed with RE.

**Table 1:** Demographic data of the patients with newly diagnosed epilepsy, *N* (%) or mean ± standard deviation.

**Variable**	**Total at start**	**Refractory epilepsy**	**Seizure free**	***P*-value^**d**^**
	**(*n* = 466)**	**(*n* = 68)**	**(*n* = 244)**	
Gender				0.142
Male	283 (61)	46 (68)	141 (58)	
Female	183 (39)	22 (32)	103 (42)	
Age of onset, y	31.2 ± 18.5	27.4 ± 16.7	31.4 ± 18.3	0.093
Duration of disease, y	3.91 ± 7.69	4.75 ± 8.75	3.42 ± 7.10	0.280
Baseline frequency of seizure per month, median (interquartile range)	1.00 (2.52)	2.75 (14.0)	1.00 (1.50)	<0.001
Lower average income (<160 USD/month)	61 (13)	14 (21)	27 (11)	0.040
Types of seizure				0.089
Focal	418 (90)	66 (97)	215 (88)	
Generalized	43 (9.2)	2 (2.9)	26 (11)	
Unknown	5 (1.1)	0 (0.0)	3 (1.2)	
History of status epilepticus	21 (4.5)	5 (7.4)	8 (3.3)	0.137
Etiology				0.212
Structural	96 (21)	19 (28)	41 (17)	
Genetic	1 (0.2)	0 (0.0)	1 (0.4)	
Infectious	9 (1.9)	1 (1.5)	5 (2.0)	
Immune	1 (0.2)	0 (0.0)	0 (0.0)	
Unknown	359 (77)	48 (71)	197 (80)	
Family history of epilepsy	49 (11)	11 (16)	24 (9.8)	0.143
History of febrile seizure	44 (9.4)	7 (10.3)	23 (9.4)	0.671
MOCA^a^^+^, score	24.1 ± 4.62	24.1 ± 4.58	24.6 ± 4.24	0.482
GAD-7^b^, score	4.62 ± 4.38	5.35 ± 4.76	4.58 ± 4.05	0.428
c-NDDI-E^c^, score	8.09 ± 3.19	8.34 ± 3.34	7.90 ± 3.02	0.444

a *MOCA, Montreal cognitive assessment*.

b *GAD-7, Generalized Anxiety Disorder 7-item Scale*.

c *c-NDDI-E, Chinese version of the Neurological Disorders Depression Inventory for Epilepsy*.

d *The p-value between the refractory group and seizure-free group*.

Comparing the demographic data between the RE group and the seizure-free group, patients with RE were inclined to having a lower average income (*Z* = −1.764, *p* = 0.078) and younger age of onset (*Z* = −1.679, *p* = 0.093). The baseline seizure frequency in the RE group was more than that in the seizure-free group (*Z* = −3.911, *p* < 0.001).

### Response to the First ASM

**Figure 2:**
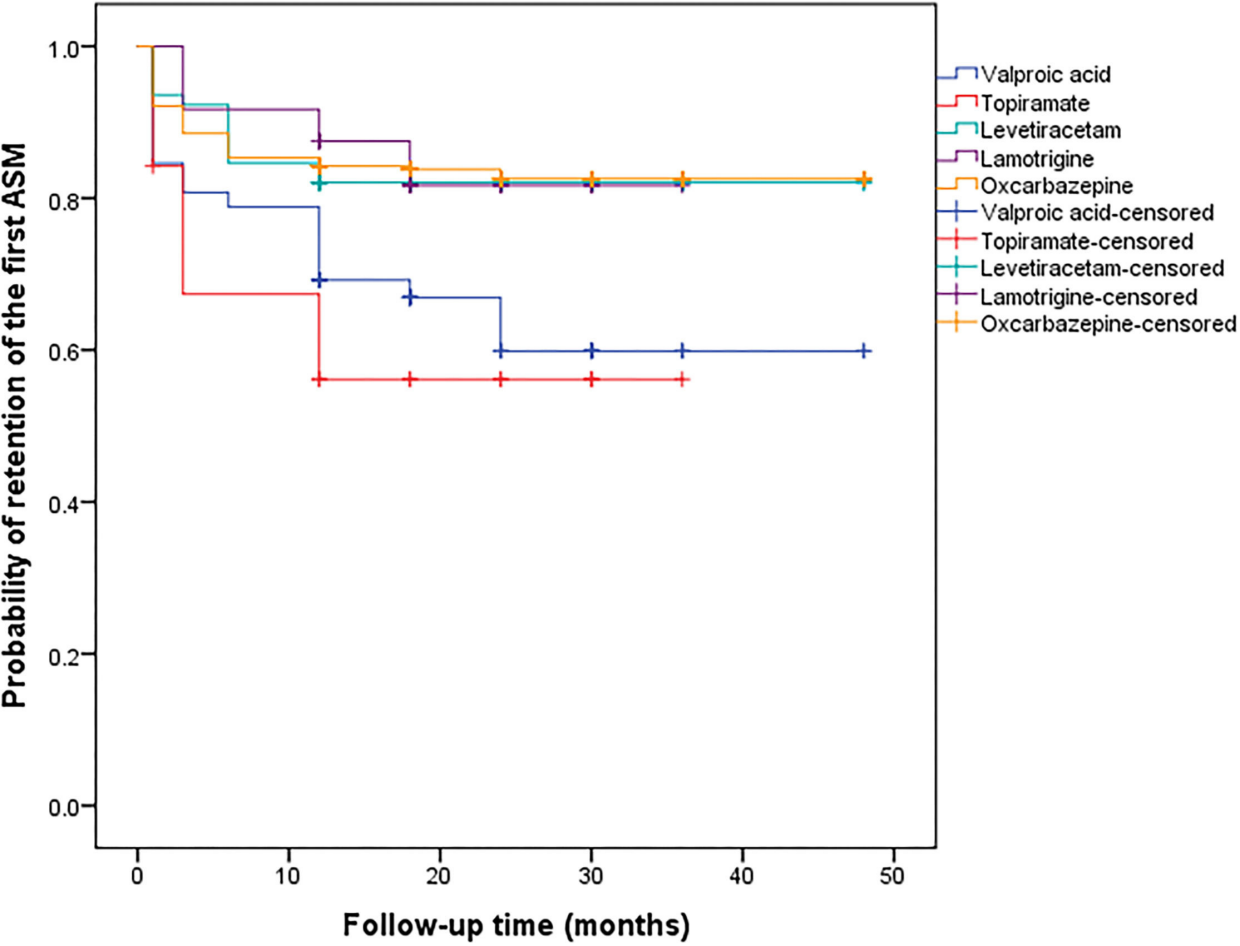
Probability of retention of the first antiseizure medication.

The first ASMs administrated to the patients are shown in Table 2. The focal seizure was the most common type of seizure and oxcarbazepine was the most commonly used ASM. A total of 370 (79%) patients remained on the first ASM at the last follow-up and 286 (61%) patients remained on the first ASM as monotherapy, among which 186 (40%) patients achieved seizure-free status. Among those who did not reach seizure-free status with the first ASM, 174 patients were treated with monotherapy (100 remaining on the first ASM with increased dosage and 74 switching to another monotherapy) and 102 patients with multiple therapy at the last visit; among these patients, 24% (*n* = 68) developed RE and 21% (*n* = 58) were seizure-free. For those who reached seizure-free status with the first ASM, the maintenance doses are shown in Table 2. The median maintenance doses were no more than 50% DDD except for oxcarbazepine. At the 12- and 24-month follow-up, lamotrigine (88 and 82%), levetiracetam (82 and 82%), and oxcarbazepine (84 and 83%) had a higher probability of retention, and topiramate had the lowest probability of retention (56 and 56%, respectively). Carbamazepine, phenobarbital, and other ASM (pregabalin and gabapentin) were excluded from the comparison due to the limited number of patients. The probability of retention of the first ASM is shown in Figure 2. There was a significant difference between the probability of the different types of ASMs (χ^2^= 17.807, *p* = 0.001). A total of 183 (39%) patients reduced the dose of the first ASM due to adverse effects, among whom 96 patients withdrew the first ASM. The causes of withdrawal or dose-reduction are shown in Table 3. The objective adverse effects were drowsiness, ataxia, dizziness, headache, memory decline, irritability, weight gain or loss, palpitation, and gastrointestinal complaints, among others.

**Table 2:** Doses of the first antiseizure medication (ASM) for patients who reached seizure-free status with the first ASM.

	**At baseline**,	**As the only**	**Seizure-free**,	**Median**,	**Maximum**,	**Minimum**,
	***n* (%)**	**monotherapy, *n* (%)**	***n* (%)**	**mg/d**	**mg/d**	**mg/d**
Valproic acid	52 (11)	20 (7.0)	14 (70)	500.0	750	400
Carbamazepine	8 (1.7)	4 (1.4)	2 (50)	500.0	800	200
Oxcarbazepine	279 (60)	186 (65)	122 (66)	600.0	1,200	240
Topiramate	19 (4.1)	5 (1.7)	2 (40)	100.0	125	50
Levetiracetam	78 (16.7)	52 (18)	37 (71)	750.0	1,250	375
Phenobarbital	4 (0.9)	3 (1.0)	1 (33)	–	–	–
Lamotrigine	24 (5.2)	17 (6.0)	8 (47)	112.5	150	100
Others^a^	2 (0.4)	1 (0.3)	0 (0.0)	–	–	–

a *“Others” refers to pregabalin and gabapentin*.

**Table 3:** Causes of withdrawal or dose-reduction of the first antiseizure medication, *n* (%).

	**Allergy**	**Ineffective**	**Liver damage**	**Other objective adverse effects**	**Poor compliance**	**Seizure-free for 3 y**
Valproic acid	3 (9.4)	9 (28)	4 (13)	9 (28)	8 (19)	1 (3.1)
Carbamazepine	0 (0.0)	0 (0.0)	1 (25)	1 (25)	2 (50)	0 (0.0)
Oxcarbazepine	19 (19)	16 (16)	24 (25)	28 (29)	10 (10)	1 (1.0)
Topiramate	3 (21)	2 (14)	0 (0.0)	6 (43)	3 (21)	0 (0.0)
Levetiracetam	1 (3.8)	7 (26)	7 (26)	9 (33)	2 (7.4)	1 (3.7)
Phenobarbital	0 (0.0)	1 (50)	1 (50)	0 (0.0)	0 (0.0)	0 (0.0)
Lamotrigine	1 (17)	3 (50)	1 (17)	0 (0.0)	1 (17)	0 (0.0)
Others^a^	0 (0.0)	0 (0.0)	0 (0.0)	1 (100)	0 (0.0)	0 (0.0)

a *“Others” refers to pregabalin and gabapentin*.

Cox regression was used to analyse the influencing factors of the retention of the first ASM. Considering the types of the first ASM, gender, age of onset, average income, disease duration, seizure frequency, and types of seizure at baseline as independent variables in the Cox regression model analysis of the first ASM retention, the hazard ratio (HR) of withdrawal of valproic acid and topiramate were 2.31 [95% confidence intervals (CI): 1.35–3.93] and 2.93 (95% CI: 1.38–6.20), respectively, compared to that of oxcarbazepine.

### Risk Factors of Refractory Epilepsy

At the last visit, 4 (0.9%) patients were receiving no ASM, and 360 (77%), 81 (17%), 18 (4%), and 3 (0.6%) patients were receiving one, two, three, and four ASMs, respectively. The ratio of seizure-free patients was 0.4% (no ASM, *n* = 1), 91% (one ASM, *n* = 223), 7.4% (two ASMs, *n* = 18), and 0.8% (three ASMs, *n* = 2), respectively. During the treatment, 83 (18%) patients had ever withdrawn the ASMs without medical advice but the ASMs were re-administered at the nearest follow-up. Approximately 24% (*n* = 16) of the patients in the RE group and 16% (*n* = 38) in the seizure-free group had poor compliance, and no significant difference was found (χ^2^ = 2.352, *p* > 0.05). Breakthrough seizures during the first expected interictal interval following ASM treatment were compared between the RE (77%, *n* = 52) and seizure-free group (26%, *n* = 63), and there was a significant difference (χ^2^ = 58.622, *p* < 0.01).

**Figure 3:**
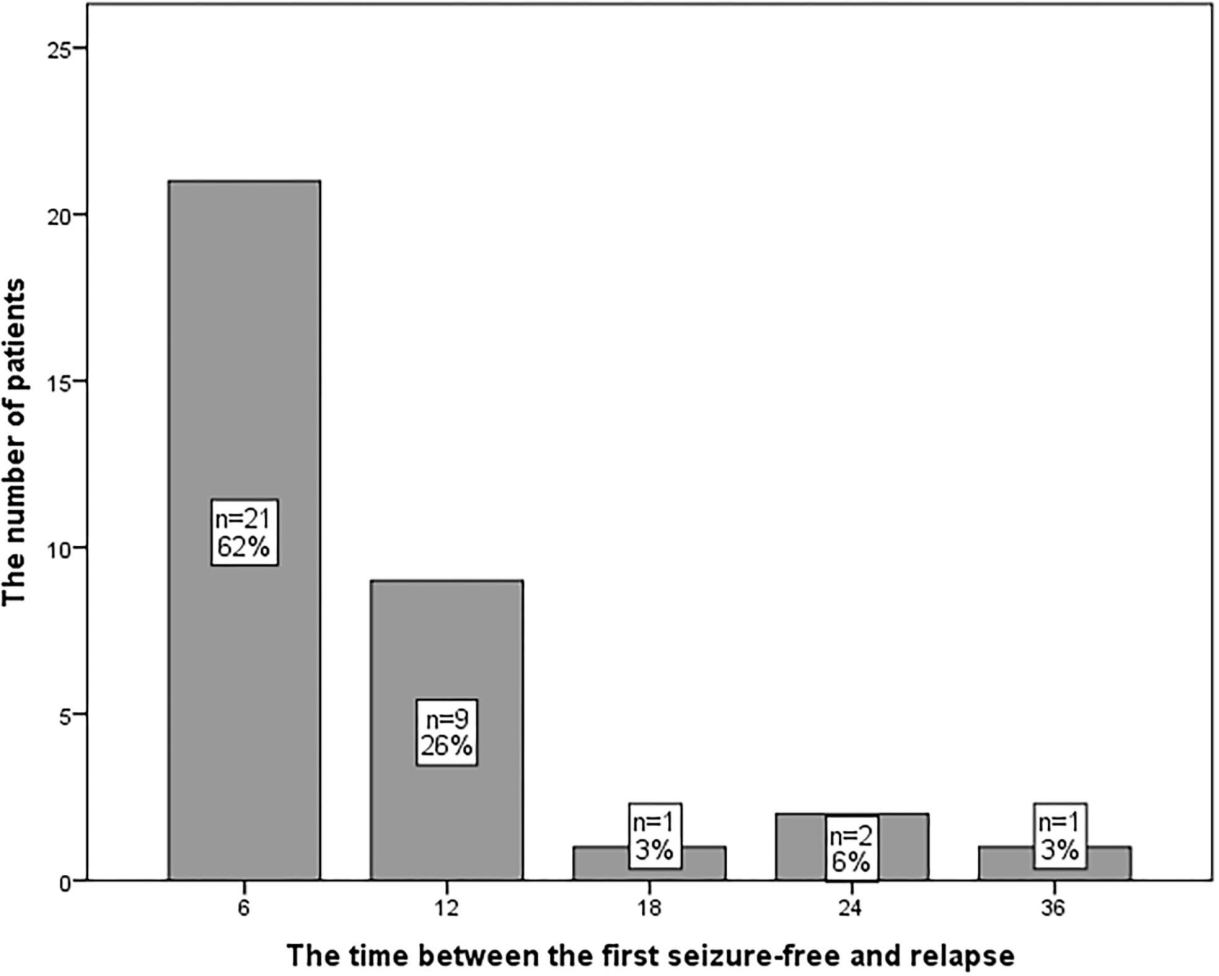
The distribution of patients with seizure relapse.

Fifteen (22%) patients who had been diagnosed with RE reached seizure-free status following ASM adjustment (they were still classified to RE in the statistics described above). Among them, six, eight, and one patient(s) were treated with one, two, and three ASM(s), respectively. No significant difference in the demographic data was found between the patients with RE who achieved seizure-free status and patients who had not (*p* > 0.05). Finding alternative effective ASMs and increasing doses of the ASM in use were methods to achieve seizure-free status. Thirty-four patients (14%) had breakthrough seizures after being classified as seizure-free. The time of relapse was 6 to 36 months (median 6 months) (Figure 3). No significant difference in the demographic data or poor compliance was found between patients with seizure relapse and those without (*p* > 0.05). One patient developed RE after identifying as seizure-free.

Logistic regression was applied to analyse the risk factors of RE, and gender, age of onset, average income, disease duration, seizure frequency and types of seizure at baseline, history of status epilepticus, etiology, compliance, and breakthrough seizures during the first expected interictal interval were set as independent variables (Table 4). Breakthrough seizures during the first expected interictal interval (OR = 5.66, 95% CI: 3.05–10.51) and higher seizure frequency (increased every 5 times/month) (OR = 1.20, 95% CI: 1.02–1.41) were risk factors. When the scores of MOCA, GAD-7, and c-NDDI-E were adjusted in the analysis, men were more likely to develop RE than women [Odds ratio (OR) = 2.66, 95% CI: 1.26–5.62], and a younger age of onset (decrease of every 10 years) (OR = 1.42, 95% CI: 1.12–1.79) was also a risk factor of RE. Meanwhile, the ORs of breakthrough seizure during the first expected interictal interval and higher seizure frequency were 5.53 (95% CI: 2.57–11.92) and 1.22 (95% CI: 1.02–1.46), respectively (Table 4).

**Table 4:** The logistic regression analysis for risk factors of refractory epilepsy in newly diagnosed epilepsy.

	**Variables**	***p*-value**	**OR**	**95% CI**	
				**Lower**	**Upper**
Before adjusted by scales	Breakthrough seizures during the first expected interictal interval	<0.001	5.66	3.05	10.51
	Higher seizure frequency (increased every 5 times/month)	0.033	1.20	1.02	1.41
After adjusted by scales	Breakthrough seizures during the first expected interictal interval	<0.001	5.53	2.57	11.92
	Higher seizure frequency (increased every 5 times/month)	0.033	1.22	1.02	1.46
	Male gender	0.010	2.66	1.26	5.62
	Younger age of onset (decrease of every 10 years)	0.003	1.42	1.12	1.79

## Discussion

ILAE published a new definition of RE in 2010 to set up explicit and practical criteria. Based on this definition, we conducted the first prospective study on treatment outcome of NDE in Northeast China, and we identified the risk factors of RE according to the new definition, which can help physicians more quickly and accurately identify patients that are likely to develop RE.

Nearly half of the adult patients with NDE became seizure-free in our study and 91% of them were treated with monotherapy. This proportion is lower than that in previous studies (15, 16), but the criteria in these studies were relatively lenient compared to the ILAE criteria (no seizures for at least the previous year). Forty percent of the patients achieved seizure-free status with the first monotherapy and the median maintenance doses were no more than 50% DDD except for oxcarbazepine. This is consistent with the conclusion of previous studies that responsiveness may be identified with exposure to low ASM doses (12, 17). Most of the seizure-free statuses were obtained by monotherapy. Although Chi et al. found that combination therapy could increase the ratio of seizure-free patients compared to monotherapy (18), the latter is more acceptable for PWE in our clinic for fear of adverse effects. Dash et al. also found that reduction of the numbers of ASM may not aggravate seizures but decrease the side effects (19). Hence, combination therapy was always applied during the period of switching to another ASM or when the monotherapy did not work in our experience.

The probability of retention and the efficacy of levetiracetam and oxcarbazepine were satisfactory as the monotherapy, and liver damage and other objective adverse effects were the main causes of withdrawal. As a traditional ASM, valproic acid had relatively lower retention but it was also very efficient. Lamotrigine had a high likelihood of retention but did not perform as well as the other drugs. Neither the retention nor the efficacy of topiramate were satisfactory, and objective adverse effects were the main cause of withdrawal. In some studies with children, lamotrigine had better retention than oxcarbazepine (20) and topiramate (21). For older adults, carbamazepine is more likely to cause withdrawal symptoms than lamotrigine, levetiracetam, and valproic acid (22). Levetiracetam, on the other hand, has better efficacy than that of lamotrigine (23). Levetiracetam and oxcarbazepine were the more favorable drugs in terms of better tolerance and efficacy in our study. Unfortunately, we could not analyse their retention in older adults due to the limited number of patients.

The incidence of RE in adult NDE in our study was 15%, which is similar to the result of the systematic review on NDE (17%) (24). Although the ILAE definition is the minimum criteria, it could take more than 1 year for the majority of the patients to identify as RE. Moreover, patients with RE were inclined to have lower income, which means that the pharmacotherapy with the possibility of poor effect would put a huge burden on this population. Timely diagnosis helps physicians and patients to consider other optimal treatments, such as resective or palliative surgery, neurostimulation (25, 26), and ketogenic diet (27).

Breakthrough seizures during the first expected interictal interval, high seizure frequency at baseline, younger age of onset, and the male sex were risk factors of RE in our study. Younger age at seizure onset and high initial seizure frequency were discussed as predictors of RE in previous studies (28–30). The breakthrough seizures during the first expected interictal interval reflect responses to the first ASM and the longitudinal data could be a more accurate predictor. Jiang et al. posited that more than two seizures in the first year after ASM initiation predicted less likelihood of achieving 2-year remission. Making the interictal interval as the observing time may be more suitable for each PWE with different seizure periods. Hughes et al. (31) found both the presence and number of post-breakthrough seizures indicated poor outcomes. Only one patient developed RE after achieving seizure-free status in our study, and others were undetermined for limited post-seizure follow-up; therefore we cannot reach the same conclusion. Previous research found that men were more susceptible to temporal lobe epilepsy-like seizures and seizure-related damage (32). Therefore, the severity of epilepsy and the degree of hemicranial volume loss were worse in men than that in women. The finding supports our conclusion that male sex was a risk factor of RE.

Nearly 14% of the patients with seizure-free status had seizure relapse and 88% of them had a relapse within 12 months. Hence, prolonging the period of ASM treatment and careful withdrawal should be emphasized, and the minimum period of ASM treatment should be 2 years of seizure-free status (33). Although diagnosing as RE, 22% of the patients achieved seizure-free status after changing to the alternative ASM regimen or increasing the doses of the ASMs in use, which is supported by a previous study (34). A patient with identified seizure-free status developed RE later in the course of her epilepsy. This is consistent with the patterns of previous research, and excessive expression of transporters for ASM removal and reduced drug-target sensitivity are the major probable theories (35). A new approach in anti-epilepsy rather than antiseizure treatment is necessary to reverse the unsatisfactory treatment scenario.

In conclusion, treatment outcomes of the majority of the NDE are good, and monotherapy could be efficient at a low dose. Levetiracetam and oxcarbazepine performed best in tolerance and efficacy. Breakthrough seizures during the first expected interictal interval, high seizure frequency at baseline, younger age of onset, and male sex predicted RE. Achieving seizure-free status is not enough to start the withdrawal of ASMs. RE is not permanent and seizure-free may be achieved subsequently by appropriate drug adjustment.

## Limitation

This was a single-center study and the findings might be difficult to extrapolate in the global settings. The follow-up period was not sufficient to determine RE for a part of patients. However, as our program is still going on, the follow-up time would be extended and the “undetermined” patients may achieve their outcome at the subsequent visits.

## Data Availability Statement

The raw data supporting the conclusions of this article will be made available by the authors, without undue reservation.

## Ethics Statement

The studies involving human participants were reviewed and approved by the Ethics Committee of The First Hospital of Jilin University [the approval number: 2017-326]. Written informed consent to participate in this study was provided by the participants' legal guardian/next of kin.

## Author Contributions

NL, JL, CC, and YC are responsible for including participants, data entry, and following up. WL is the designer of this project. All authors contributed to the article and approved the submitted version.

## Funding

The work was supported by fund from the Clinical Research Development Fund of The First Hospital of Jilin University [fund number: lcpyjj2017006].

## Conflict of Interest

The authors declare that the research was conducted in the absence of any commercial or financial relationships that could be construed as a potential conflict of interest.

## Publisher's Note

All claims expressed in this article are solely those of the authors and do not necessarily represent those of their affiliated organizations, or those of the publisher, the editors and the reviewers. Any product that may be evaluated in this article, or claim that may be made by its manufacturer, is not guaranteed or endorsed by the publisher.
